# Mechanical, Physico-Chemical and Morphological Characterization of Energy Optimised Furnace (EOF) Steel Slag as Coarse Aggregate in Concrete

**DOI:** 10.3390/ma15093079

**Published:** 2022-04-24

**Authors:** Arivoli Masilamani, Malathy Ramalingam, Parthiban Kathirvel, Gunasekaran Murali, Nikolai Ivanovich Vatin

**Affiliations:** 1 Department of Civil Engineering, Sona College of Technology, Salem 636005, India; malathycivil@sonatech.ac.in; 2 School of Civil Engineering, SASTRA Deemed University, Thirumalaisamudram, Thanjavur 613401, India; 3 Peter the Great St. Petersburg Polytechnic University, 195251 St. Petersburg, Russia; murali_22984@yahoo.com (G.M.); vatin@mail.ru (N.I.V.)

**Keywords:** steel slag, chemical composition, physical property, mechanical property, scanning electron microscope, digital image processing

## Abstract

This research tests energy optimised furnace (EOF) steel slag as substitution for natural coarse aggregate in concrete. Steel slag’s usefulness as a substitute for natural coarse aggregate in concrete is the primary goal of this research. According to IS:2386-1963, the characterization of EOF steel slag, as coarse, is done by examining the shape and size of a particle, mechanical properties, physical properties, soundness, and alkali-aggregate reactivity. Tests for detection of staining material in steel slag and hardness of inter-facial transition zone in hardened cement paste were also carried out. The chemical analysis of the steel slag reveals the stability of oxides present in the steel slag. Microstructural characterization by SEM (scanning electron microscope) analysis of steel slag aggregate was also employed to support the characterization and XRD analysis, and it was found that the EOF steel slag is crystalline. The digital image processing technique (DIP) is adopted to study the shape indices, circularity, sphericity, shape factor, and roundness of natural and EOF steel slag aggregate. According to the characterization and strength investigation, steel slag aggregate outperforms natural coarse aggregate.

## 1. Introduction

Concrete is a versatile composite material used for the worldwide constructions that is made up of coarse granular material known as aggregates that are strongly linked together with the assistance of cement to form a strong structure. The quality of aggregate used therein is the major contributing factor to the quality of concrete; proper proportioning and aggregate mixing influence the concrete strength and mechanical properties [1]. As the mechanical forces are received and conducted by the aggregate, they can be referred to as the skeleton of the concrete. Raw steel slag is produced as a by-product in the process of steel-making during the change of iron into steel [2]. In a blast furnace, molten slag and steel are formed by oxidation of molten iron, metals, scrap, and flux to remove impurities [3]. The slow cooling of molten slag converts it into a dense rock-like material. As per the Indian scenario, the steel industry generates a huge quantity of waste, which will increase enormously with increasing steel production. According to the Ministry of Environment and Forests and Climate Change (MOEFCC) and Central Pollution Control Board, India, steel slag is produced at a rate of 4 million tons annually, which are continuously discarded in open spaces. A typical blast furnace slag is produced at the rate of 300–400 kg per tonne of liquid iron produced in India. The quantity of steel slag produced has increased from 35.35 million tonne (out of 101.4 million tonne of steel production) in 2017–2018 to 53 million tonne (out of 150 million tonne of steel production) in 2019–2020, which tends to strengthen the utilization of 100% slag effectively and economically viable [4].

Nippon slag association [5] has been developing technology to resolve difficulties related to the consumption of steel slag. The mining, transportation, and processing of natural resources will be reduced if steel slag is utilized in place of natural aggregate. Additionally, there will be less disruption to the land, less energy consumption, less pollution of air, land, water, and less emission of greenhouse gases. Reducing the amount of natural aggregate in the concrete mix by replacing it with steel slag can enhance the sustainability of natural resources, since the extraction and transportation phases in the production of natural aggregate are eliminated. Steel slag, a waste product, forms the process of steel-making, requires a little energy to produce. The steel industry will be benefited, since their by-products will be used beneficially, instead of degrading the environment by unsafe dumping. The American National Slag Association (NSA) [6] established that iron and steel-making slag poses no health or environmental hazards. The great mechanical resistance of steel slag makes it superior to natural aggregate when used as an aggregate. Steel slag as a filler ingredient in concrete has been the subject of several investigations.

It has been identified that the free calcium oxide (CaO) present in the steel slag is the dominant cause of expansion as a result of the formation of calcium hydroxide and calcium carbonate when reacted with water [7]. By minimizing the free calcium oxide content in steel slag, the stress resulting from the expansion of free calcium oxide will be lowered and higher will be the stability of cement concrete. Steel slag undergoes mineral change at high temperatures, resulting in irregular volumetric expansion that reflected adversely on slag-based concrete mixtures’ residual properties [8]. The harmful effect of free CaO could be minimized to acceptable limits by aging. During this aging, the steel slag is stockpiled for 4–6 months and hydration of free lime (CaO) takes place with atmospheric moisture. Aging for a long time mainly reduces the presence of harmful expansive materials in steel slag [9]. By studying the morphological character of and electric arc furnace steel slag and basic oxygen furnace (BOF) slag, it can be seen that slag particles have high sphericity, solid structure, and favorable frictional properties [10]. The scanning electron microscope test reveals the steel slags rough texture, which enhances the mechanical strength of concrete [11].

In building road foundations and anti-slippery blacktop, steel slag may be substituted for the limestone aggregates entirely to generate lightweight concrete, due to its hydraulic property [12]. The compressive, split tensile, and flexure strength were enhanced by between 4 and 8 percent in concrete made using steel slag as coarse aggregate, compared to the control mixes. The density of concrete is increased by 5 to 7% when using coarse aggregate that is 100 percent slag, rather than natural aggregate, as a reference [13]. Recent researchers reported that the split tensile, flexural, and compressive strength of steel slag aggregate concrete were higher than the control mix [7,14,15]. This shows that adding slag could satisfy the role of the accelerator at the initial stage, whereas, at 28 days, the effect diminished. Replacement of slag notches is the primary outcome [16]; additionally, the utilization of iron slag in concrete can augment the concrete strength properties [17]. Steel slag aggregate concrete has also inclined to decrease the water absorption characteristics and porosity after 28 days, compared to the reference mix with coarse aggregate in the form of stone chips [14].

The aggregate shape can profoundly characterize the aggregate quality, affecting concrete mixtures property. Daily, measuring the shape parameter using calliper devices is tiresome and time-consuming for quality control. Digital image processing (DIP) has been applied to characterize aggregate shape in recent years. The relationship between the DIP and experimental results was strong. It was inferred that the DIP method was a much faster and improved substitute for traditional particle shape measurement [18,19,20]. The surface roughness, shape, and surface area of fine aggregate were analyzed by data acquisition procedures [20]. The shape features of the aggregates were examined using Image J, including elongation, flatness, roundness, aspect ratio, form factor, shape factor, and sphericity. The coarse aggregate’s shape and texture greatly influenced the concrete slump. As the sphericity and form factor of aggregate increased, the compressive strength also increased [21,22]. Interfacial roughness, specific surface area, and aspect ratio of the lightweight aggregate significantly affected mechanical strength [23].

Many investigations have been conducted to see whether concrete aggregate may be replaced with industrial by-products. It is observed from the previous studies that the characteristics of steel slag largely depend on the production process of steel and the chemical composition of flux added [6,8,9]. EOF steel slag is a by-product of steel production industries. EOF steel slag is not used in cement production because it has no cementitious properties. The mechanical qualities of EOF steel slag are critical to its use, and they are intimately linked to concrete’s physical, chemical, and morphological features. The chemical composition of the EOF steel slag influences the volumetric stability of the resulting material. Similarly, the EOF steel slag based concrete strength is a function of the mechanical properties of steel slag. This paper presents a thorough characterization of EOF steel slag to facilitate its use in concrete. In this context, an experimental investigation has been carried to evaluate the effectiveness of EOF steel slag on the fresh properties (such as the slump cone test), hardened properties (such as compressive strength), microstructural characteristics (such as scanning electron microscope), X-ray diffraction, and shape parameters (using digital image processing of concrete mixes) were carried out, and the results are summarized.

## 2. Material Properties

### 2.1. Cement and Sand (Fine Aggregate)

According to IS 8112-2013 [24], 43 grade ordinary Portland cement (OPC) was employed as a binder during the whole investigation. A sieve test was used to assess the cement fineness and observed that the residue on 90 µ sieve was 8%, which is less than 10%, as required for OPC. The cement has a specific gravity, standard consistency, and initial setting time were 3.16, 31%, and 35 min, respectively. The fine aggregate used in this investigation has been acquired from a riverbed, which has the advantage of a cubical shape with a smooth surface. The fine aggregate physical properties used in this investigation are detailed in Table 1, which satisfies the requirement of IS 383-2016 [25], since they have a more significant impact on the fresh concrete properties.

### 2.2. Coarse Aggregate and EOF Steel Slag

Construction-grade aggregate, made from locally sourced granite, was utilized to produce concrete. Aggregates of less than 20 mm in size were crushed and well-graded by machines. Steel-making operations produce this slag as a by-product when pig iron and steel scrap are transformed into steel. For its outstanding toughness and workability, this steel is highly prized. It is known that approximately 200 kg of slag is created for every ton of steel production. In JSW Steel Ltd., Salem Works (JSWS), 12,000–13,000 tons of energy optimized furnace (EOF) slag is produced per month and 200,000–300,000 m^3^ of EOF slag is produced per annum. The utilization of steel slag in concrete delivers excellent mechanical properties.

### 2.3. Test Techniques of Coarse Aggregates

**Figure 1:**
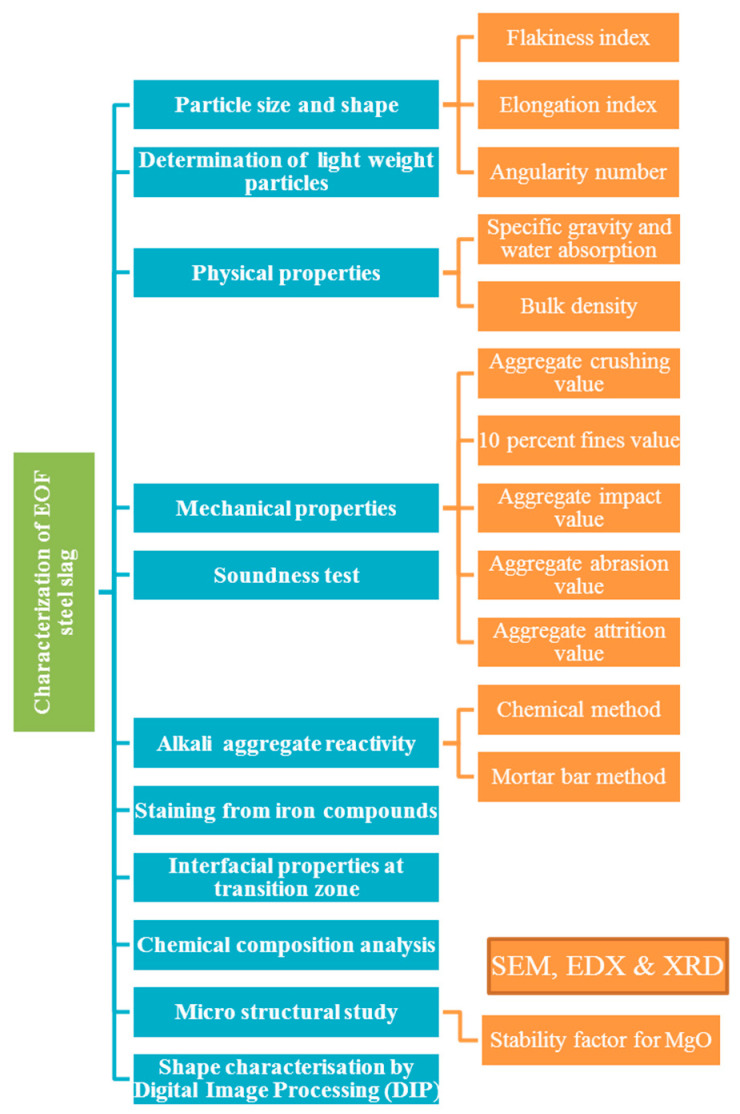
Framework of characterization study.

Scarcity in aggregate availability from natural sources is being faced in many situations. This problem can be overcome by using natural aggregate as minimum as possible by replacing it with industrial by-products, such as EOF steel slag. As aggregates are essential components for making concrete, and their properties are greatly affected by various characteristics of the aggregate. For this reason, it is vital to investigate the materials characteristics that are utilized in concrete production. This knowledge will help in choosing appropriate ingredients for good concrete quality. EOF steel slag characterization requires the following tests, and these tests were employed according to ASTM C641-98 [26], ASTM C123-03 [27] and IS: 2386:1963 [28,29,30,31,32,33]. A comparison is made between the methods and outcomes and the characteristics of natural coarse aggregate. Figure 1 discusses the systematic research framework used to characterize steel slag for use as coarse aggregate in concrete step-by-step.

#### 2.3.1. Particle Size and Shape

A suitable gradation of aggregate is a significant aspect of producing workable concrete, which contains all standard aggregate fractions in the required proportion. There are minimal voids that need little paste to fill the spaces between the aggregate in graded aggregates. It was necessary to reduce the nominal size of the raw and aged slag from steel mills to 20 mm, in order to make it easier to include in concrete mixes. Using sieve analysis, the gradation of EOF steel slag was determined according to IS 383-2016 [25], which indicates the coarse aggregate for concrete. The aggregate shape is an imperative characteristic because it dramatically influences the workability and consolidation of fresh concrete. EOF steel slag shape properties, such as the flakiness index, elongation index, and angularity number, are evaluated. With a greater length to thickness ratio than the specified values, the particles are elongated and flat. The flat and elongated particles tend to break easily, causing adverse effects in concrete. An aggregate’s angularity, or the sharpness of its particles, is an essential feature, since it affects how easily an aggregate mixture may be handled. This supports strong interlocking, binding hardened concrete and stimulates workability of concrete at fresh state. The stability of a concrete mixture relies on the degree of particles interlocking.

#### 2.3.2. Determination of Light Weight Particles

Lightweight particles affect durability and tend to cause pop-outs. Therefore, it is essential to determine the presence of lightweight particles by following the ASTM C 123 [27] guidelines. Zinc chloride salt was diluted in a sufficient quantity of distilled water, in order to get the required specific gravity of 2.4 for the solution. EOF steel slag sample was allowed to submerge in the prepared zinc chloride solution.

#### 2.3.3. Physical Properties

All aggregates are porous, and the amount of liquid absorbed by an aggregate, when submerged in water, depends on its porosity. Aggregates water absorption value impacts water–cement ratio and fresh concrete workability [34]. EOF steel slag has a water absorption rate of around 3%, much greater than that of natural aggregate. This means that the EOF steel slag must be saturated before being mixed with concrete, in order to lower its water absorption capability.

#### 2.3.4. Mechanical Properties

It was considered that concrete is an amalgamation of separate particles held together by a cementing agent. The concrete’s strength is determined by the mechanical characteristics of aggregate, regardless of the cement paste quality and bonding with aggregate. According to IS: 2386 (Part IV)—1963 [32], experiments were conducted on a EOF steel slag and natural coarse aggregate to determine their mechanical properties, including crushing, 10% fines, impact, and the Los Angeles abrasion value. Several factors contribute to concrete’s strength, including mechanical properties. Aggregates that are strong and durable are necessary for producing strong concrete.

#### 2.3.5. Soundness Test

For concrete, the aggregates’ resistance to the effects of weathering or their long-term durability is a significant consideration. In order to determine aggregate durability, sodium sulphate soundness testing is one of the most often used methods. When an aggregate is subjected to a chemical assault, it is utilized to calculate the percentage loss of the aggregate, according to IS: 2386 (Part V)—1963 [32]. The aggregate was sorted into size fractions: fractions crossing 20 mm retained 10 mm, fractions passing 10 mm retained 4.75 mm, and fractions passing 10 mm kept 4.75 mm. At a temperature of 23 °C, the aggregate sample was subjected to a sodium sulphate saturated solution. The aggregate fractions were subjected to 10 immersion cycles in the salt solution, with each cycle requiring 16 h of immersion time in the solution. Following each immersion cycle, 105 °C oven was used to dry the samples.

#### 2.3.6. Alkali Aggregate Reactivity (AAR)

Chemical reactions of alkalis present in concrete and some alkaline minerals present in aggregate, are termed alkali aggregate reactivity (AAR). Alkali-silicate gel formed from AAR attracts moisture by osmosis or absorption, which increases volume, due to the surrounding confinement, internal pressure results, causes of the disruption of cement paste, and cracking of aggregate. There are two methods to determine alkali aggregate reactivity. One is the chemical method in which this test was performed, in order to identify the presence of reactive minerals in the aggregates that reacted with the alkalis present in the cement. Another method is the mortar bar method. Through the measurement of the expansion created in mortar bars, this test seeks to determine the potential expansive alkali reactivity of a cement-aggregate mixture. IS: 2386 [33] standard procedure was followed to cast, cure and store mortar bars of dimension 25 × 25 × 250 mm appropriately. For this test, the coarse aggregates were crushed into fine aggregates to meet the requirements.

#### 2.3.7. Staining from Iron Compounds

Unsightly stains appeared in exposed concrete surfaces, due to the occasional presence of iron oxide and iron sulfide particles in aggregates. As per ASTM C641 [26], the test to know the presence of any insoluble iron components in steel slag was carried out. The presence of iron compounds in aggregate attributes the potential degree of staining, which is evaluated primarily using a visual classification method. If the aggregate has insoluble products from the decomposition of iron components, the insoluble products will deposit as black, red, and green strains.

#### 2.3.8. Interfacial Transition Zone

Normal cement concrete has a weak point at the interfacial transition zone between hardened cement paste (HCP) and aggregate, roughly 100 µm wide. To further understand the performance of the HCP aggregate interface and difference between the steel slag aggregate HCP interfaces and natural coarse aggregate, micro-hardness variation in this area was evaluated. The micro-hardness was determined by standard Vickers hardness testing equipment. The hardness is defined as the ratio of applied load and area of the indentation. The test was performed on the surface of concrete specimens polished with emir sheets and tested with a pyramid diamond cone indentor of apical angle of 136°. It is necessary to measure the diagonal length of the indentation for a particular force and then use conventional tables to calculate the hardness. Alternatively, the following formula may be used to determine hardness.

Hardness (*HV*) = (1854.4 × *P*)/*D*^2^ (1)

where, *D* is the mean diagonal length (mm) and *P* is the applied load (g).

#### 2.3.9. Microstructural Study

Energy dispersive X-ray (EDX), SEM, and XRD analysis studied the microstructure properties of EOF steel slag. The microscopic examination of steel slag particles revealed their shape and surface texture. EOF steel slag particles were examined with a SEM (Jeol JSM 6390 scanning electron microscope). Elemental analysis was performed using EDX analysis.

#### 2.3.10. Stability Factor for MgO

Free MgO hydrates take months or even years to form, resulting in a considerable volume change. The slag generated from molten steel making technology has low MgO content [6]. The hydration of MgO causes the autogenous expansion, forming Mg(OH)_2_ crystals, which are larger than the sum of volumes of MgO and H_2_O (IS: 2386 (Part V)—1963) [32]. The presence of unstable MgO content can be confirmed by determining the stability factor ‘K’.

Stability factor K = MgO/(FeO + MnO)(2)

if K < 1 MgO is stable or K > 1 MgO is unstable

#### 2.3.11. Shape Parameters from Digital Image Processing

The shape aggregate can profoundly characterise the quality of aggregate, as it affects concrete mixtures property. Daily, measuring the shape parameter using calliper devices is tiresome and time-consuming for quality control. Digital image processing (DIP) has been applied to characterise aggregate shape in recent years. Four indices are chosen to characterize natural aggregate and EOF steel slag: sphericity, circularity, shape factor, and roundness (angularity index).

Sphericity is expressed as the ratio of the surface area of a sphere whose volume is as that of aggregate to the original surface area of aggregate [19]. To find sphericity, the following Equation (3) [35] is used:

(3) $$\mathrm{Sphericity}=\sqrt[{3}]{\frac{\mathrm{bd}}{l^{2}}}$$

where, b is breadth area of particle, d is depth of the particle, and l is the length of the particle.

Circularity is expressed as the ratio between the perimeter of the equivalent circle of aggregate and perimeter of the aggregate. If the circularity is one, the shape of the aggregate is smooth and round [19]. Therefore, if the circularity is zero means, the shape of aggregate is less smooth and round. The circularity of the particles is calculated by Equation (4):

(4) $$\mathrm{Circularity}=\sqrt{\frac{4\pi A}{P^{2}}}$$

where, *P* is the perimeter of the particle, and *A* is the projected area of the particle.

Roundness is a measure of sphericity; the roundness value of a particle closer to one means the particle is more circular in shape [19], i.e., for a circle, the roundness value is 1. The higher roundness of an aggregate indicates lesser fracture faces on the aggregate surface [20]. Roundness is measured by the given formula [36]:

(5) $$\mathrm{Roundness}=\frac{P^{2}}{4\pi A}$$

Shape factor of aggregate is a dimensionless quantity that defines the aggregate shape. The shape factor is independent of the size of the aggregate. The ranges of the shape factor are 0–1. The following equation proposed by Kuo et al. (1996) [36] is used to calculate the shape factor.

(6) $$\mathrm{Shape}\;\mathrm{factor}=\frac{d}{\sqrt{\mathrm{bl}}}$$

A computerised technique to capture and digitalize the scene is a two-dimensional image expressed as digital image processing (DIP). A scanner or a video camera can be used to capture the scene. The produced video signals are stored as pixels; later, they are analysed to generate the required information. Various algorithms and techniques can be engaged to generate the required information. In this study, the DIP technique is applied in concrete technology to investigate aggregate shape parameters.

**Figure 2:**
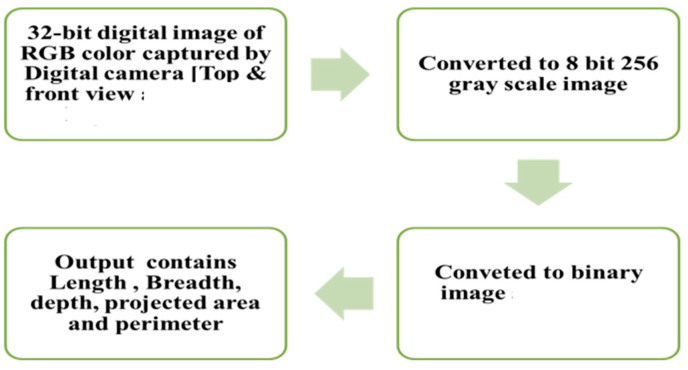
Image processing methodology of DIP.

The algorithm adopted identifies shape variations in greyscale or in colour of an aggregate to define the edges of the aggregate and discriminate the aggregate form the background. Many public domain programs are available in DIP technique. During this investigation, Image J is employed as an image analysis application. The National Institute of Health in United States has developed Image J, a Java-based program for image processing. Image J can be downloaded in public domains. The working methodology of image processing is represented in Figure 2.

#### 2.3.12. Slump Test

Fresh concrete workability is essential as the concrete needs to be adequately compacted, transported, readily placed, and adequately finished. A concrete mix having good workability will produce concrete with maximum density and less void space. An increase in the density of concrete improves the concrete strength. To measure the workability of concrete, a slump test is commonly carried out. The mix proportion for different water–cement ratios was arrived at by following the recommendations of IS: 10262-2009 [37] standard. Mild exposure circumstances and high-quality standards have been included in the design. Different mix proportions were used, i.e., 1:1.94–3.21, 1:1.45–2.61, and 1:1.42–2.62 ratio of cement, fine aggregate, and coarse aggregate for water–cement ratios of 0.55, 0.45, and 0.44, respectively.

#### 2.3.13. Compressive Strength

The compressive strength of hardened concrete is a primary mechanical property to confirm whether the concrete has the requisite specified strength. Several factors, i.e., water–cement ratio, aggregates’ grading, size, surface texture, shape, and superplasticizer dosage, influence the concrete strength. With EOF steel slag and natural coarse aggregate, 150 mm cubic concrete cubes were made. Twenty-four hours afterward, the specimens were de-molded and exposed to a 28-day water curing procedure. The compressive strength of all specimens was tested and evaluated, as per IS: 516-1959 [38].

## 3. Discussion of Results

### 3.1. Particle Size and Shape

**Figure 3:**
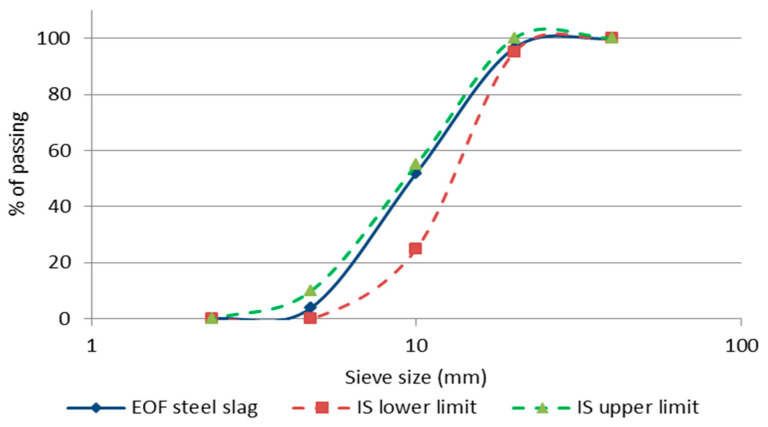
Granulometric curve.

Figure 3 depicts the particle size distribution for a graded aggregate with a nominal size of 20 mm, which is then compared to the limitations of percentage passing established by IS 383-2016 [25] for this aggregate. Table 2 demonstrates the properties of EOF steel slag and natural coarse aggregate. Compared to natural coarse aggregate, the EOF steel slag has a lower flakiness index, suggesting that the slag particles are thicker. Because EOF steel slag has a lower elongation index than natural coarse aggregate, it may be inferred that slag particles are shorter and rounder than the natural coarse aggregate. The natural coarse aggregate’s flakiness and elongation index value is 33.2%. EOF steel slag is 24.4%, which should not exceed 40% for concrete mixes and 30% for bituminous non-bituminous mixes, as recommended in IS 383-2016 [25]. The lesser percentage of flat particles makes sure high stability in concrete. According to Table 2, EOF steel slag (20.83) and natural coarse aggregate (20.41) have the same angular number. EOF steel slag’s angular and rougher surface texture will ensure a better degree of interlocking with cement matrix [39]. It can be observed from Table 2 that the EOF steel slag particles are thicker than natural coarse aggregate, and the angular and rougher surface texture of EOF steel slag will ensure a better degree of interlocking with the cement matrix.

### 3.2. Evaluation of Light Weight Particles

**Figure 4:**
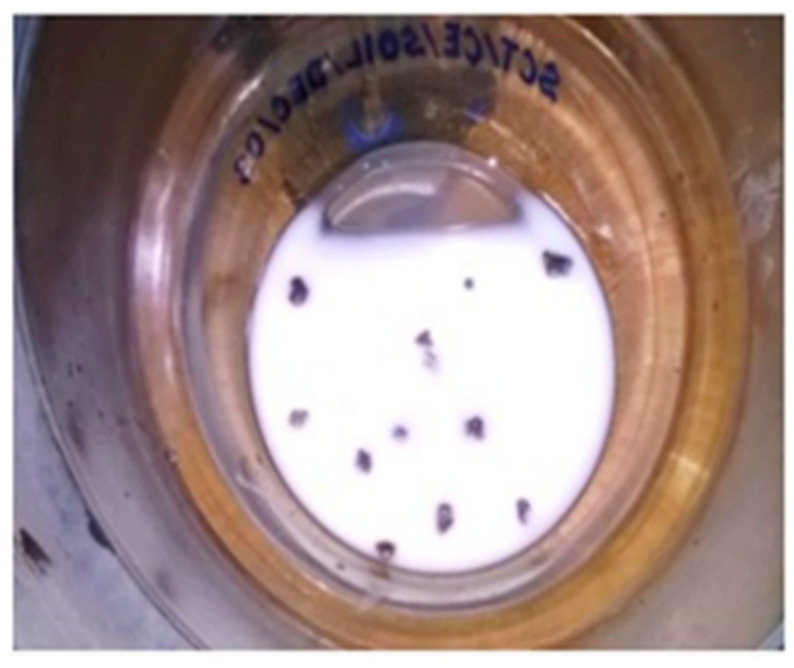
Lightweight particles floating in the zinc chloride solution.

As shown in Figure 4, the lightweight particles in the EOF steel slag sample began to float on the solution’s surface. The floating fraction is collected separately. The collected fraction of the sample is dried in the oven for 24 h and is weighed. The percent of lightweight particles is found to be 3%. This meets the prescribed limit of 3%. The effects of lightweight particles in concrete could be assessed by studying the durability properties of concrete. There are no traces of other deleterious materials, such as clay, coal, lignite, and particles, finer than 75 µm in the EOF steel slag. Therefore, the sample taken meets the limit of total deleterious content of 5%, as per IS: 383—2016 [25].

### 3.3. Physical Properties

According to the criteria established in IS 2386 (part III) (1963), the physical characteristics of EOF steel slag and natural aggregate are investigated and shown in Table 3 [30]. EAF slag has a bulk density 4.6 percent higher than natural coarse aggregate, which is likewise the case with EOF steel slag [40]. Steel slag has a denser composition than natural coarse aggregate, with a density difference of 4% [34], due to high iron content [39]. Natural aggregates have a lower bulk density and specific gravity (see Table 3) than BOF and EAF steel slag (see Table 3).

### 3.4. Mechanical Properties

Table 4 shows the mechanical parameters of EOF steel slag. For the strength tests, such as the crushing, 10% fines, and impact values, steel slag has greater values than natural aggregate. EOF steel slag has a greater crushing value because it is more resistant to crushing under progressively applied compressive stress. IS: 2386 [32] states that EOF steel slag may be utilized in concrete pavement wear surfaces and other concrete constructions because of its strong crushing strength of above 30%. The EOF steel slag’s increased impact value results from its resistance to failure under sudden loading, as well as the greater the concrete’s elastic modulus, due to the stronger aggregate [41]. Resistance against fragmentation of EOF steel slag aggregate is 7.11% higher in the Los Angeles abrasion test [34] and 14.88% higher in Deval’s attrition test (resistance against wear) than that of the natural aggregate. The impartial resistance to wear and fragmentation of EOF slag particle qualifies its use as a replacement for natural aggregate in high strength concretes (50–60 MPa) [40].

### 3.5. Soundness Test

**Figure 5:**
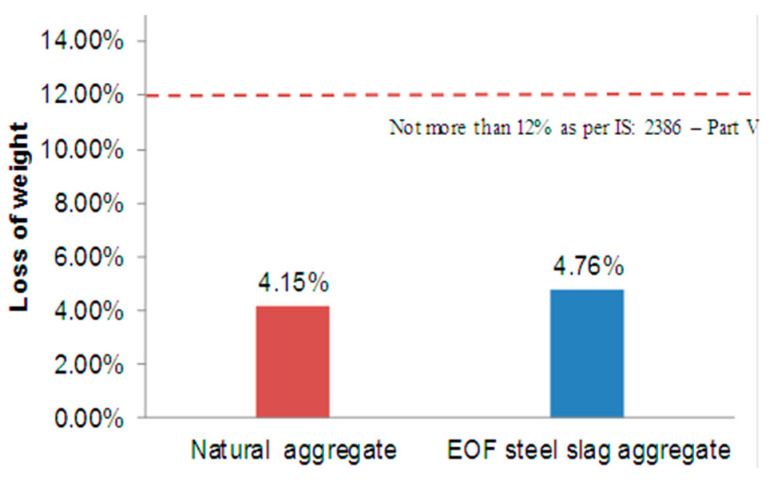
Percentage losses in soundness test.

Figure 5 shows the results of the sodium sulphate soundness test for natural aggregate and EOF steel slag. Soundness is the resistance against volume change brought out by changes in physical condition. The EOF steel slag has non-hydrated lime and magnesium oxides derived from the steel-making process. The free calcium and magnesium are susceptible to expansion when exposed to moisture. The soundness test result is presented in Figure 5 and points out that the resistance of EOF steel slag against volume change is comparable to natural aggregate. This can be attributed to the aging process happening in the EOF steel slag during its storage. The EOF steel slag is left as a stockpile over 4–6 months. During this aging process, free lime and magnesium react with atmospheric moisture and are converted into magnesium hydroxide (Mg(OH)_2_) and calcium hydroxide (Ca(OH)_2_). Throughout the aging, the steel slag is free to expand in this aging process. The expansion of EOF steel slag is considerably minimized in this aging process; thereby, EOF steel slag becomes free from expandable materials when used in concrete [40].

### 3.6. Determination of Alkali Aggregate Reactivity

**Figure 6:**
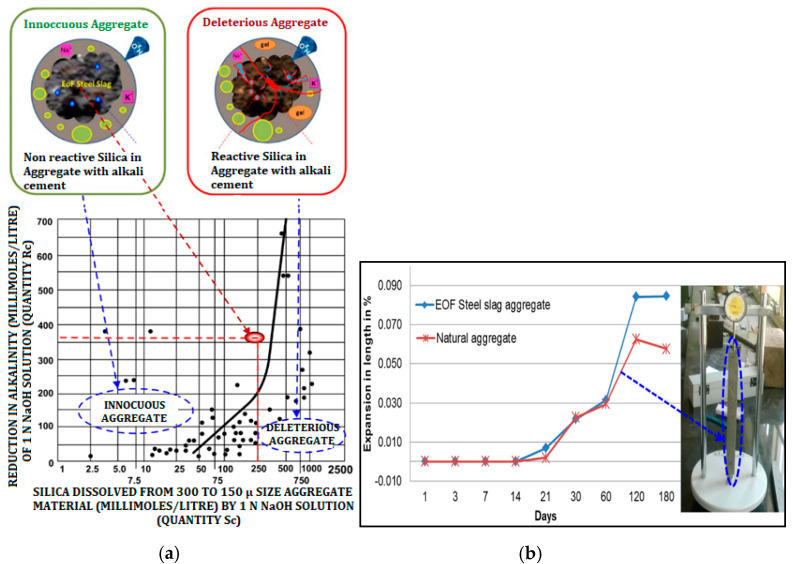
Alkali aggregate reactivity of EOF steel slag (**a**) chemical method with chart (IS: 2386 (part VII)—1963); (**b**) mortar bar method.

The chemical method’s test results of alkali reactivity are incorporated in the graph of IS: 2386-1963 [33], as shown in Figure 6a. Figure 6a also represents the non-reactivity mechanism of EOF steel slag with alkali cement. The points lie to the left of the boundary line. It indicates that EOF steel slag causes mortar expansion of less than 0.1 percent in a year, under the same conditions. The EOF steel slag can very well be considered an innocuous aggregate. By the mortar bar method, the difference in the length of the mortar bars was recorded for 6 months. The percentage of change in length of bars is displayed in Figure 6b. It is observed from the figure that there is no expansion at the early ages (i.e., from day 1 to 7); from 7th day onwards, the expansion is reflected in an increase in length of all specimens. Beyond 30 days, the increment in the expansion percentage of natural aggregate is low, when compared to progressive increment in EOF steel slag aggregate. The permissible limits for the expansion of the mortar bar test at 38 °C are 0.05% at 90 days and 0.10% at 180 days, which are recommended by ASTM C641-98 [26]. Though the EOF steel slag aggregate meets the permissible limits, it is expansive in nature, compared to natural aggregate.

### 3.7. Staining from Iron Compounds

**Figure 7:**
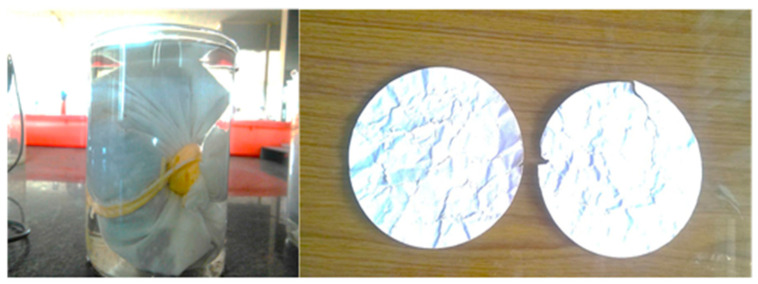
Filter papers without staining.

Figure 7 shows the filter paper before and after being subjected to a steam bath. It is observed from Figure 7 that there are considerably no visible strains in the filter papers. From this, it is surmised that there is no decomposed iron component in the EOF steel slag. The absence of stain in the filter paper also proves that there are no soluble iron components present, which may tend to avoid the unpleasant appearance on the surface of concrete. In addition, the absence of soluble iron components tends to reduce the transformation of ferrous components to ferric components, thereby reducing the effect of the corrosion of the concrete reinforcement.

### 3.8. Properties of the Interfacial Transition Zone

**Figure 8:**
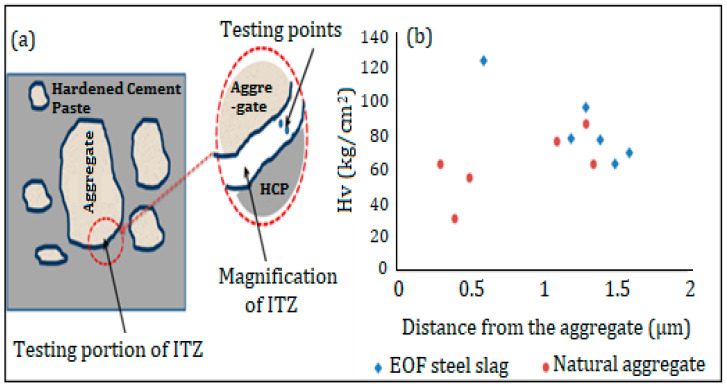
(**a**) Interfacial transition zone between hardened cement paste (HCP) and aggregate, (**b**) Micro hardness value of interfacial transition zone.

Normal cement concrete has a weak point at the interfacial transition zone between the hardened cement paste (HCP) and aggregate, which is roughly 100 µm wide. Figure 8 shows that the microhardness was tested in the interfacial transition zone [41] as shown in Figure 8a. The results of micro-hardness are shown in Figure 8b, and it is evident that the hardness value of EOF steel slag aggregate HCP is greater than that of natural coarse aggregate. It is inferred that the hardened cement paste gets consolidated, due to hydration of EOF steel slag, i.e., the addition of steel slag to concrete increases the chemical reaction. Furthermore, the steel slag’s rough surface texture improves the concrete’s strength because of its strong adhesion [42].

### 3.9. Stability Factor for MgO

**Figure 9:**
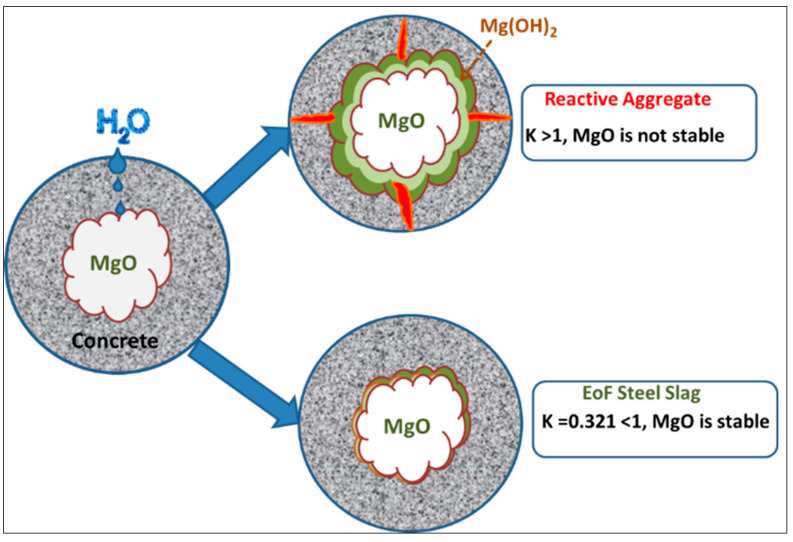
Stable Mechanism of Magnesium Oxide in EOF steel slag.

By substituting the values of oxides from Table 5, in Equation (2), the value of stability factor K is determined as 0.322, which is less than one. This indicates that MgO exists in solid solution with FeO and MnO. Therefore, it is deduced that MgO is stable. Figure 9 shows the non-reactivity mechanism of EOF steel slag with stable MgO. The reactive aggregate will expand by forming Mg(OH)_2_ and forming cracks because of K > 1, but EOF steel forms cracks because K < 1.

### 3.10. Microstructural Study

**Figure 10:**
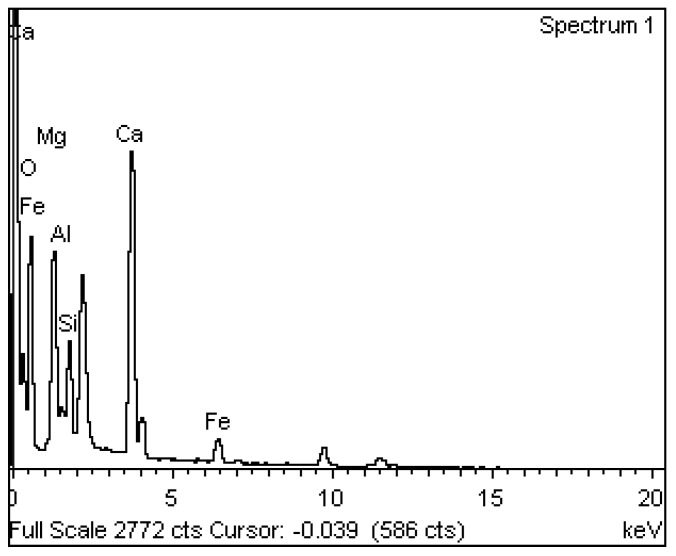
EDX spectrum of EOF steel slag.

The EDX spectrum of the EOF steel slag sample that confirms the presence of oxides of Ca, Mg, Al, Si, and Fe appears in Figure 10. In general, the chemical composition of steel slag is mainly variable, depending upon materials, process, and location [45].

**Figure 11:**
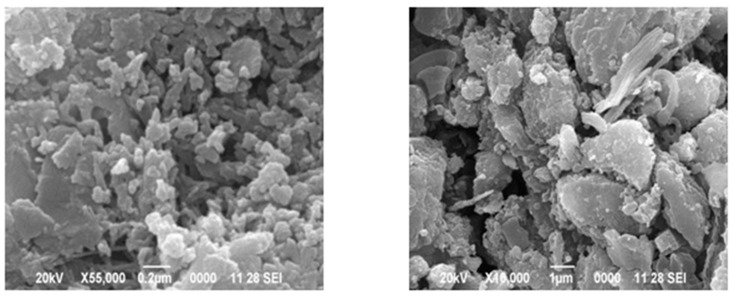
SEM micro graph of EOF steel slag, (**a**) magnification ×55,000, and (**b**) magnification ×100,000.

**Figure 12:**
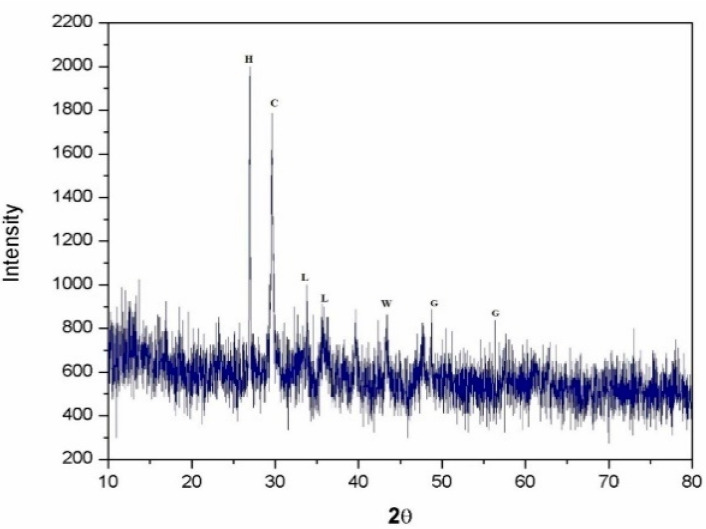
X-ray diffraction analysis of EOF steel slag.

Figure 11a,b shows the morphology of EOF steel slag. The microstructure of EOF steel slag exhibits subrounded and angular shapes [10,43]. Most of the particles are examined using SEM, which showed extremely crystalline structures and rough textures with platy. The platy particles examined by SEM are highly irregular in shape. This morphology provides a greater surface area to form a strong transition zone between the aggregate and hardened cement paste. The EOF steel slags chemical analysis is presented in Table 5, and the percentage of chemical composition is compared with other types of steel slag, such as EAF steel slag. The major chemical oxides identified are CaO, MgO, SiO_2_, and Al_2_O_3_ [11,34]. The amount of MnO is insignificant. In transforming molten pig iron into steel, a certain percentage of iron (Fe) remains as hot metal, and this fraction cannot be streamlined into the produced steel. Chemical investigation of steel slag samples reveals the presence of this oxidized iron [44]. Because so much lime (flux) is needed in the iron-to-steel conversion process, EOF steel slag often has a high CaO component [11,43]. Figure 12 shows the XRD pattern of the EoF steel slag. The diffraction pattern of EOF steel slag shows the presence of hematite-Fe_2_O_3_ (H), calcite-CaCO (C), lanite-Ca_2_SiO_2_ (L), gehlenite- Ca_2_Al(AlSiO)_7_ (G), and wusite-FeO (W). The high crystalline nature of EOF steel slag shows its weak potential for any hydraulic activity that confirms dimensional stability. EOF steel slag does not have any pozzolanic activity, due to its high crystallinity [45]. This is highly attributed to the EOF steel slag’s volume stability, which is major quality of aggregate [46,47].

### 3.11. Shape Parameters from Digital Image Processing

**Figure 13:**
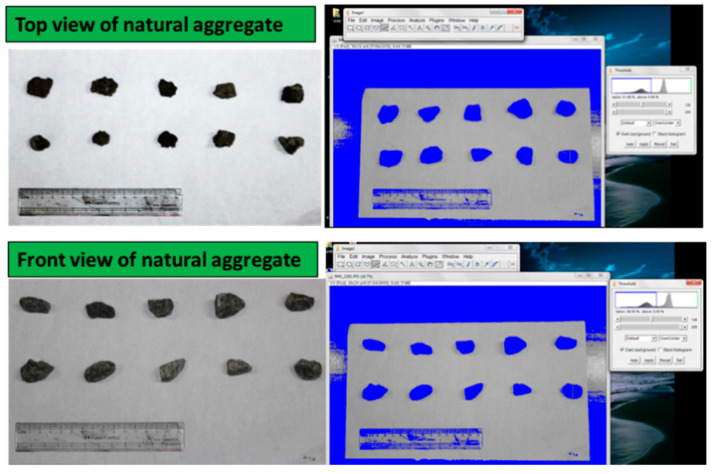
Top and front view of natural aggregate and thresholded images.

**Figure 14:**
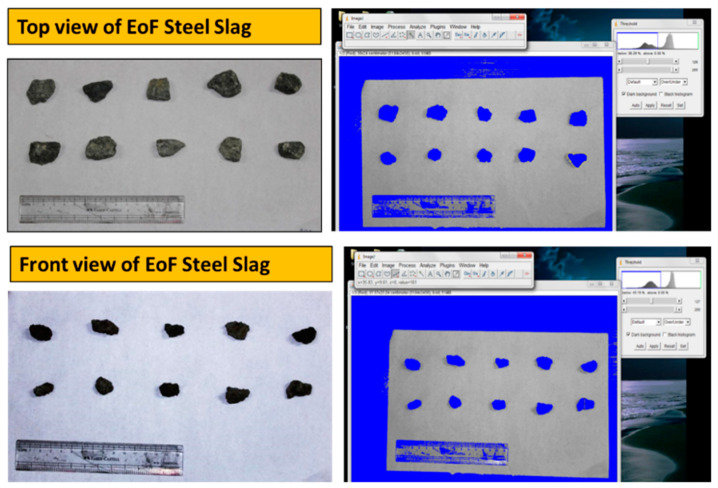
Top and front view of EOF steel slag and thresholded images.

**Figure 15:**
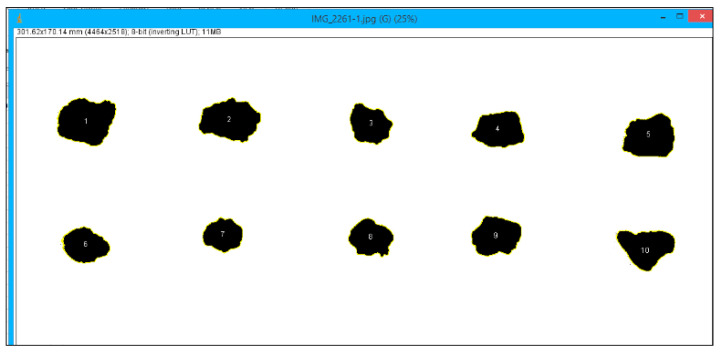
Binary image of aggregate with numbering.

Image of natural aggregate and EOF steel slag are captured using a high-resolution digital camera. Eighty particles from each natural aggregate and EOF steel slag are chosen. The particles are placed on a white background to capture the top and front views. Then, the images are processed through Image J. The virtual quality of the original image is enhanced, and the particles are highlighted. Later, the chosen particles are segmented from the background by choosing the appropriate threshold value. Figure 13 and Figure 14 show the natural aggregate and EOF steel slag’s graphical and threshold image. In segmenting particles having lower intensity, threshold values are set to blue when the background is white. Figure 15 represents the binary image of the aggregate sample with numbering.

The shape parameters of 80 particles each from matural coarse aggregate (NCA) and steel slag aggregate (SSA) are noted. Statistically, the results of the shape parameter obtained from DIP are significant with a 95% confidence interval (high precision). Table 6 gives the arithmetic mean of the shape parameters of natural aggregate and EOF steel slag. From the Table, it is inferred that the circularity of EOF steel slag is lesser than the natural coarse aggregate, which indicates EOF steel slag is less smooth and highly irregular in shape than the natural coarse aggregate. Sphericity and shape factor explains the form (complete shape) of the aggregate [19]. The sphericity and shape factor of EOF steel slag is marginally higher than natural coarse aggregate and comparable to natural coarse aggregate. The mean value of sphericity and shape factor shows that the natural coarse aggregate and EOF steel slag are non-spherical and highly angular. The mean roundness value indicates that the natural aggregate and steel slag aggregate are of other shapes. It also denotes that EOF steel slag aggregate has an irregular surface and rough edges, since the roundness of EOF steel slag is lesser than that of natural aggregate. From the lesser roundness value of the EOF steel slag, it can be inferred that the EOF steel slag has more fractured faces. More fractured faces mean more surfaces contact with cement paste, which ensures better interlocking of aggregate [20].

### 3.12. Workability of Concrete with EOF Steel Slag

For a moderate environment, IS 456: 2000 [48] recommends that the degree of workability of concrete should be in the range of 50 mm to 100 mm of a slump. From Table 7, it is perceived that irrespective w/c ratio, all concrete mixes with EOF steel slag meet the least slump value of 50 mm. The EOF steel slag aggregates with irregular shapes and rough surfaces necessitate more cement volume to cover the surface than natural aggregates require [39,49]. The decrease in the workability is highly significant in the lower water–cement ratio because of higher water absorption [44,50].

Another factor is the higher water absorption capacity of EOF steel slag. As the slump value lies within 50 mm, EOF steel slag can be utilized as a partial substitution/replacement for natural coarse aggregate in normal and heavily reinforced concrete, with compaction and uniform dispersal through manual vibration [9,48].

### 3.13. Compressive Strength of EOF Steel Slag Based Concrete

**Figure 16:**
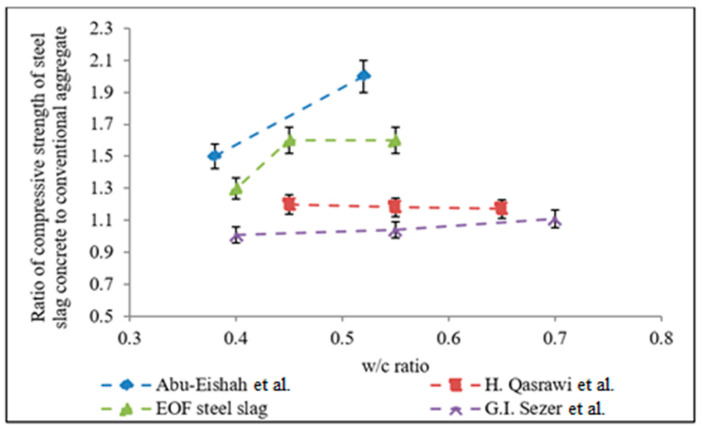
Concrete compressive strength versus water–cement ratios.

Figure 16 shows a comparison of the compressive strength of concrete containing EOF steel slag and natural aggregate. The specimens comprising of EOF steel slag as a coarse aggregate exhibited a significant improvement in compressive strength, compared to the specimens comprising of a natural coarse aggregate at the same water–cement ratio. This is attributed to rough surface roughness, which promotes good adhesion between EOF steel slag the cement matrix. Interfacial transition zone integrity is an important factor in determining concrete’s strength [44]. The hydration of CaO in the EOF steel slag has resulted in a thick, continuous, and hardened transition zone in the aggregate concrete. Aside from the physical contribution, the formation of cement paste surrounding EOF steel slag is facilitated by the hydration of EOF steel slag [6,9,51]. In addition, the EOF steel slag’s Los Angeles abrasion value is lower than natural aggregate. The lesser abrasion value means a higher aggregate strength, which enhances a compressive strength of concrete [11]. Figure 11 shows that the enhancement of the compressive strength of concretes with water–cement ratios of 0.55, 0.45, and 0.4 were 15%, 22%, and 12%, respectively.

## 4. Conclusions

The current research was employed to compare the size of particle, shape, physical, mechanical, and chemical properties of EOF steel slag with conventional natural aggregates. Different ranges of graded EOF steel slag aggregate sizes facilitate good packing in concrete. The shape properties of EOF steel slag aggregate are more or less angular, making the workable concrete. Lightweight particles are present in EOF steel slag in insignificant quantities. The EOF steel slag had a greater water absorption capability than natural aggregate, which made the concrete more difficult to work. The mechanical properties show a remarkably better performance of EOF steel slag, in terms of crushing, impact, abrasion, and attrition. The soundness test and AAR test show the absence of expandable materials; additionally, there is no trace of decomposition of iron compounds in EOF steel slag. Steel slag aggregate concrete has a higher microhardness than natural aggregate because of its rough-textured surface and chemical reactivity with the cement matrix. From the chemical composition, it is noted that magnesium oxide is stable and inexpensive. The DIP analysis shows that EOF steel slag is considerable, similar to the natural aggregate, in the aspects of better form (sphericity and shape factor), roundness, and circularity. EOF steel slag aggregate morphology gives rise to a stronger interfacial transition zone, due to its rough-textured surface. The hardened interfacial zone and rough surface texture of EOF steel slag contributes to enhancing concrete strength.

## Figures and Tables

**Table 1:** Physical properties of Fine aggregates.

Description of Physical Properties	Obtained Value
Bulk density	1450 kg/m^3^
Surface moisture	Nil
Fineness modulus	3.1 (zone II)
Water absorption	1.57%
Specific gravity	2.6

**Table 2:** Shape properties of natural coarse aggregate and EOF steel slag.

Test	EOF Steel Slag	Natural Coarse Aggregate
Angularity number	20.41	20.83
Elongation index	7.7%	15%
Flakiness index	16.7%	18.2%

**Table 3:** Physical properties of coarse aggregate.

Test	Natural Coarse Aggregate	EOF Steel Slag	EAF Steel Slag [34]	BOF Steel Slag [39]	EAF Steel Slag [39]	EAF Steel Slag [40]
Specific gravity	2.75	2.86	3.58	3.26	3.64	3.68
Bulk density (kg/m^3^)	2886	3020	2581	2049	2092	3810
Water absorption	0.8%	3%	0.7%	2.31%	1.75%	2.4%

**Table 4:** Mechanical properties of coarse aggregate.

Type of Test	EOF Steel Slag	Natural Aggregate	Recommended Value, as per IS: 2386
Deval attrition	3.68%	18.56%	-
Los Angeles abrasion	1.16%	8.27%	Not exceeding 50%
Impact value	29.57%	22.87%	Less than 45%
10 percent fines value	8.3%	7.8%	7.5% to 12.5%
Crushing value	8%	6.55%	Not exceeding 45%

**Table 5:** Chemical composition of steel slag.

Chemical Compounds	EOF Steel Slag	EAF Steel Slag [11]	Steel Slag [43]	EAF Steel Slag [34]	Steel Slag [44]
CaO (%)	35.28	39.62	46.73	32.52	24.62
MgO (%)	9.27	9.23	6.27	4.56	-
SiO_2_ (%)	16.69	17.23	14.77	17.17	17.79
Al_2_O_3_ (%)	6.20	4.08	5.52	7.96	7.82
MnO (%)	1.88	0.70	2.76	3.8	-
Fe_2_O_3_ (%)	26.91	20.40	18.42	30.8	35.3
P_2_O_5_ (%)	1.43	-	1.67	0.58	-
Na_2_O (%)	0.16	-		0.16	0.74
K_2_O (%)	0.03	-		0.03	-
SO_3_ (%)	0.56	-		0.25	0.13

**Table 6:** Shape indices of natural coarse aggregate and EOF steel slag.

Description	Natural Coarse Aggregate		EOF Steel Slag Aggregate	
	Mean	Standard Deviation	Mean	Standard Deviation
Circularity	0.692	0.0844	0.627	0.1078
Sphericity	0.992	0.122	1.034	0.0985
Shape factor	0.759	0.1136	0.770	0.1264
Roundness	0.679	0.239	0.602	0.4026

**Table 7:** Workability of concrete comprising EOF steel slag.

	w/c Ratio	Slump (mm)
EOF steel slag	0.55	72
EOF steel slag	0.45	63
EOF steel slag	0.4	89
Abu-Eishah et al.	0.52 & 0.38	190 & 220
B. Pang et al.	0.42	185
Manso et al.	0.6	70
S. Brand et al. (BOF & EAF)	0.56	76 & 32
G. I. Sezer et al.	0.40, 0.55 & 0.7	110, 100 & 95

## Data Availability

Not applicable.
